# Identifying the Neurogenetic Framework of Crohn's Disease Through Investigative Analysis of the Nucleotide-binding Oligomerization Domain-containing Protein 2 Gene Mutation

**DOI:** 10.7759/cureus.5680

**Published:** 2019-09-17

**Authors:** Md Sakibuzzaman, Syed Ahmad Moosa, Mahabuba Akhter, Ipsita Hamid Trisha, Khandokar A Talib

**Affiliations:** 1 Internal Medicine, Sir Salimullah Medical College, Dhaka, BGD; 2 Family Medicine, Woodhaven Medical Professional Corporation, Queens Village, USA; 3 Biology, Southern Arkansas University, Magnolia, USA; 4 Neurology, Sir Salimullah Medical College, Dhaka, BGD; 5 Medicine, Sylhet Mag Osmani Medical College and Hospital, Sylhet, BGD

**Keywords:** crohn’s disease, nod2, card15, inflammatory bowel diseases

## Abstract

Among several inflammatory bowel diseases, Crohn’s disease is associated with inflammation that may take place in any region of the gastrointestinal tract. The inflammatory process is most commonly associated with the ileum, often spreading deep into the bowel tissues, extending into multiple forms, such as strictures and penetrations. Currently, Crohn’s disease has no known cure. Various medical and surgical procedures are used to manage the condition. The underlying mechanisms of the disease are yet to be identified, with recent studies suggesting the influence of genetics, environmental factors, and the possible activity of pathogens. Newer studies also offer strong evidence that suggests a relationship between Crohn’s disease and the nucleotide-binding oligomerization domain-containing protein 2 (NOD2) gene, also known as inflammatory bowel disease protein 1 (IBD1) or caspase recruitment domain-containing protein 15 (CARD15). NOD2 is responsible for the mechanism in which the immune system identifies foreign microorganisms through the sensing of pathogen-associated molecular patterns in microorganisms. NOD2 can detect intracellular muramyl dipeptide (MDP) in the bacterial wall, thereby causing an inflammatory response. Three major mutations associated with the NOD2 gene are known to have an influence on Crohn’s disease (SNP8, SNP12, and SNP13). This article will discuss a number of studies to identify whether there is a relationship between Crohn’s disease and the NOD2 gene.

## Introduction and background

Crohn's disease, first described by Dr. Burrill B. Crohn and colleagues in 1932, affects the entirety of the gastrointestinal (GI) tract, causing inflammation, swelling, and irritation [1]. Although Crohn’s disease can affect multiple regions of the GI tract, the most commonly affected part is the ileum, extending from the jejunum up to the ileocecal valve. Crohn’s disease is categorized as one of the chronic idiopathic inflammatory diseases [2]. According to Dahlhamer et al.’s 2016 study, the disease had affected three million American citizens by 2015, with a greater susceptibility among adults over 45 years of age [3]. A systematic review by Molodecky et al. found that Crohn’s disease was associated with an annual incidence of 3 to 20 cases per 100,000 [4]. Inflammation caused by Crohn’s disease has been established as transmural, affecting the entire wall of the gastrointestinal tract, with biopsies often demonstrating the presence of noncaseating granulomas. The disease is associated with a number of symptoms that can vary, including diarrhea, fever, pain in the abdomen, nausea, and vomiting. Crohn’s disease exhibits many phenotypes; often, a patient may even progress from one phenotype to another. This phenotypic switching generally starts with the inflammatory process and progresses to either stricturing or penetrating forms. Crohn’s disease has no known cure, with patients requiring at least one single surgical resection [5]. The precise pathogenesis of Crohn’s disease remains unknown; however, research suggests a variety of environmental and genetic factors associated with increased risks of Crohn’s disease [6]. This article will discuss the genetic relationship between Crohn’s disease, more specifically, the genetic relationship between Crohn’s disease and the nucleotide-binding oligomerization domain-containing 2 (NOD2) gene. The NOD2 gene functions as a synthesizer of NOD2 protein, which plays a significant role in the immune system function. The NOD2 protein is present in different immune cells, such as monocytes and macrophages, which sense peptidoglycan in bacterial cell walls and stimulate a host response. NOD2 mutations are associated with several inflammatory diseases, such as graft versus host disease, Blau syndrome, and Crohn’s disease. This type of response reinforces the role that NOD2 gene has in inflammation and host-pathogen interaction [7].

## Review

NOD2 gene regulatory response mechanism

With each passing moment, the human body comes into frequent and continuous contact with a plethora of microorganisms, including pathogens and commensals. In such cases, the immune system acts as the initial line of defense against multiple microbes. However, the dysfunction of the innate immune system can cause infections, as well as inflammation, in addition to autoimmune diseases. The immune system identifies foreign microorganisms through the sensing of pathogen-associated molecular patterns present on and within microorganisms. This is done by a finite number of germline-encoded pattern recognition receptors (PRR), which may exist in either the surfaces of the host cells or inside the intracellular compartments [8]. NOD2 is a cytosolic receptor belonging to the NOD-like receptor family which can detect intracellular muramyl dipeptide (MDP) in the bacterial wall [9]. MDP is available in all bacterial peptidoglycans [10]. NOD2 triggers a pro-inflammatory response when activated. In patients with chronic inflammatory diseases, including Crohn’s disease, NOD2 alterations or mutations have been identified [11-16].

**Figure 1:**
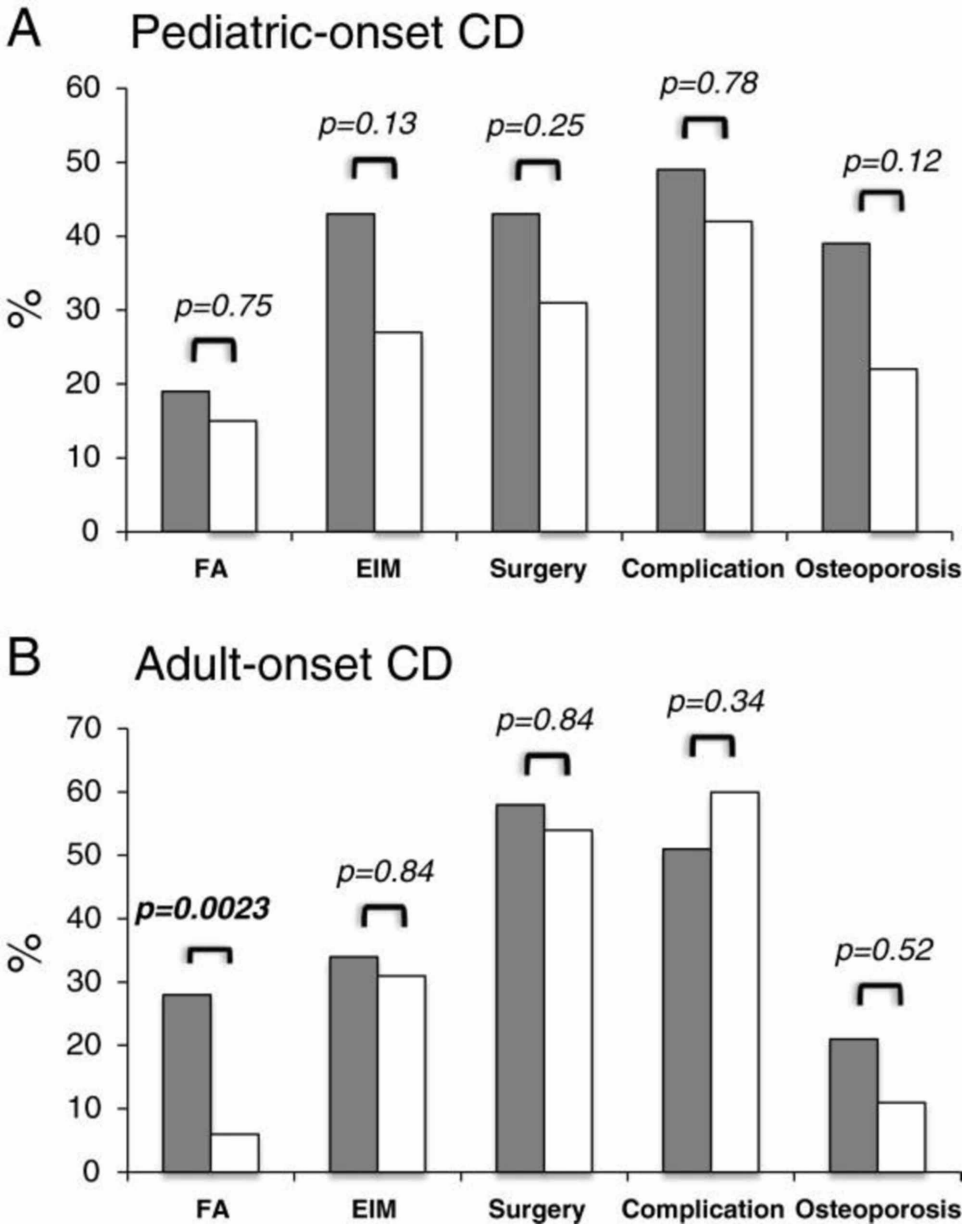
The effect of NOD2 gene variants in Crohn’s disease (CD) patients (pediatric and adult) on disease behavior Grey bars represent the NOD2 variant, and white bars represent the NOD2 wild-type alleles. Both are compared with a family history (FA) of intestinal bowel disease, extraintestinal manifestations (EIM), complications, or surgery underwent for Crohn’s disease. A p-value less than 0.05 is considered significant [24].

Recent studies have found a strong interrelationship between the NOD2 gene, previously known as caspase recruitment domain-containing protein 15 (CARD15), and Crohn’s disease. During the beginning of 2001, NOD2 was identified as the first susceptibility gene for Crohn’s disease. This theory was first presented by two independent studies [11-12]. Furthermore, in conjunction with these two studies, the consensus among researchers places NOD2 heterozygote alterations with a doubled risk for Crohn’s disease, as well as around 20 times the risk for Crohn’s disease in NOD2 homozygotes [17-22]. The NOD2 gene interacts with the lipopolysaccharide portion of bacteria, which ultimately induces cellular activation. Yet, the mechanism of Crohn’s disease via NOD2 mutation is not entirely understood. Sidiq and colleagues have identified that the NOD2 gene product is present in large quantities among Paneth's cells in the ileum; this coincides with the most commonly affected region in Crohn’s disease [23]. A study by Cleynen et al. identified that the age of onset, surgical history of intestinal bowel disease, or extraintestinal manifestations have little or no association, whereas NOD2 has a potential role in the pathology of Crohn’s disease based on the family history and disease location in the gastrointestinal tract [24] (Figure 1).

Genetic mutations fostering bowel disease

The action of NOD2 and its receptor for bacterial pattern recognition offers a valuable link to inflammatory bowel diseases. Approximately 20% of the genetic susceptibility of Crohn's disease is related to three main mutations (SNP8, SNP12 missense mutations, and SNP13 frameshift mutation) of the NOD2 gene [25]. The analysis of mutations found the NOD2 mutant to lack the last 33 amino acids, resulting in a low nuclear factor kappa B (NFκB) activity [11]. An alternative explanation is that an inborn hypo-responsiveness is a direct result of genetic mutations causing elevated NFkB from an unidentified bacterial lipopolysaccharide (LPS), inducing anti-inflammatory cytokines.

Scudiero et al. conducted a clinical trial on 42 subjects between the ages of 11 - 69 years with a mean age of 26.6 years [26]. All subjects were diagnosed with Crohn’s disease, with the control group consisting of 65 healthy adults who were not related. The results were in line with another study showing a strong relationship between NOD2 and Crohn’s disease [27]. Existing epidemiological and linkage research demonstrates a strong relationship between Crohn’s disease and genetic alterations [28-29]. The most common gene associated with the disease is the NOD2 gene [30-31]. The protein function of the NOD2 gene is reduced from polymorphism, which causes an imbalance in the inflammation responses to external stimuli [30, 32]. One allele mutation of NOD2 increased the risk of Crohn’s disease up to two to four times, and in instances of a double mutation, the risk was increased up to 20 to 40 times [33]. Lakatos et al. utilized clinical data obtained from a questionnaire completed by the physician to study the correlations between Crohn’s disease and NOD2 mutations [34]. The study was conducted among 70 Hungarian males and 72 females with a mean age of 36.2 years, and the results showed that the NOD2 mutations were 29.6% more prominent in patients diagnosed with Crohn’s disease compared to the controls. The study also found that colonic location was affected lesser than the ileal location in patients with the mutation, although the mutation was not influential on the disease characteristics. Lakatos et al. concluded that a higher amount of NOD2 mutations (specifically, the R702W and 3020insC mutations) were present in Hungarian Crohn’s disease patients. This finding further suggests that Hungarian patients were less susceptible to G908R mutations. When mutations were prevailing in the Hungarian patients, ileal disease was more common with fibrostenosis and extraintestinal diseases were rare.

There is an increased risk of being diagnosed with Crohn’s disease if a patient has a family history of the disease. Around 10 - 25% of patients diagnosed with inflammatory bowel disease have a first-degree relative who also has the disease [6]. When geographical backgrounds are considered, the same study identified that the disease is less frequent in African-Americans and Hispanics, whereas Ashkenazi Jews are more susceptible compared to non-Jews. Some clinical studies aimed at identifying the relationship between NOD2 and Crohn’s disease in twins show a concordance rate of 20 - 50% in monozygotic vs. 10% in dizygotic twins [35-37].

According to data presented by Hugot et al., four out of 10 Crohn’s disease patients are carriers one of the three NOD2 variants [38]. It is important to note here that around 15% of healthy individuals carry one of the NOD2 variants as well. Other replications may also exist, such as in IBD2, IBD3, and IBD4, for Crohn’s disease to develop. Additionally, such areas may also contain NOD2 genes, which demonstrate the possibility of additional factors to be also present (such as interactions with other genes) in addition to NOD2 defects. Such findings prove that a significant amount of additional work is needed to benefit from the relationships identified from NOD2 and Crohn’s disease-related studies. Although many studies have identified the potential role of NOD2 in Crohn’s disease, multiple other factors can also contribute to increased risks and act as different pathways for Crohn’s disease. These factors include various environmental factors, autophagy, epithelial functions, and adaptive immunity [24, 39]. Therefore, the present times are still too early to obtain a noteworthy benefit for clinicians or patients. However, continuous research in the field can offer promising outcomes and possibly a successful cure for Crohn’s disease.

## Conclusions

Crohn’s disease can affect the entire gastrointestinal tract in any region. The disease is generally managed medically, and at times, patients may require surgical intervention. Although the precise etiology of the disease is unknown, the mechanism of the disease has been identified as a result of a combination of environmental and genetic factors. Strong evidence suggests a relationship between Crohn’s disease and the NOD2 gene (or CARD15), which is situated on chromosome 16. Combining the results from multiple linkages, clinical, and sequencing studies, it can be concluded that many factors ranging from environmental factors, as well as genetics (particularly, mutations in the NOD2 gene, such as SNP8, SNP12 missense mutations, and SNP13 frameshift mutations), may significantly increase the risk of acquiring the disease. 
